# Factors influencing physical inactivity status among chinese pregnant women: a cross-sectional study

**DOI:** 10.1186/s12889-022-14757-7

**Published:** 2022-12-09

**Authors:** Tianchun Zhou, Yuping Lin, Feng Xu, Xiaoxia Ma, Na Wang, Yan Ding

**Affiliations:** 1 Nursing Department, Obstetrics and Gynaecology Hospital of Fudan University, No. 128 Shenyang Road, Shanghai, 200090 China; 2 School of Nursing, Fudan University, Shanghai, China

**Keywords:** Physical activity, Exercise, Pregnancy, Pregnant women, Factors

## Abstract

**Background:**

Regular prenatal physical activity provides numerous health benefits to both mother and fetus. However, little is known about the physical activity status of pregnant women in China and whether they meet the current guidelines for prenatal physical activity. The aims of the study were to assess physical inactivity status and associated factors among pregnant women in Shanghai, China.

**Methods:**

A cross-sectional study of 1636 pregnant women were recruited at a tertiary obstetrics and gynecology hospital in Shanghai. Maternal sociodemographic characteristics and health information were obtained using structured questionnaires or from the electronic medical records. Physical inactivity status was assessed using the International Physical Activity Questionnaire-Short Form. Factors pertinent to physical inactivity were identified by binary logistic regression and were reported with adjusted odds ratios (ORs) and 95% confidence intervals (CIs). All statistical analyses were performed using the SPSS software package.

**Results:**

In total, the prevalence of physical inactivity was 47.5%. Walking was the main form of physical activity and only 2.8% of the pregnant women achieved the goal of at least 150 min of moderate-intensity physical activity weekly. Multivariate logistic regression identified a significant negative association of physical inactivity with personal monthly income (adjusted OR 0.648, 95% CI 0.505–0.831), engagement in regular exercise before pregnancy (adjusted OR 0.575, 95% CI 0.464–0.711) and in the second (adjusted OR 0.534, 95% CI 0.411–0.693) or third (adjusted OR 0.615, 95% CI 0.470–0.806) trimester of pregnancy. Women with nausea or vomiting during pregnancy were more likely to be physically inactive during pregnancy (adjusted OR 1.307, 95% CI 1.002–1.705).

**Conclusion:**

Physical inactivity is highly prevalent among pregnant women in China. Further efforts should be taken to overcome the barriers to prenatal physical activity and to promote moderate- to vigorous-intensity activities among Chinese pregnant women.

## Background

Physical activity (PA) is defined as any bodily movement produced by skeletal muscles that requires energy expenditure, and is an essential element of a healthy lifestyle [1]. A growing body of evidence suggests that regular prenatal exercise has health benefits for both mother and fetus [2]. Maternal benefits of being active during pregnancy include control of gestational weight gain [3], less risk of gestational diabetes mellitus (GDM) [4], improved cardiovascular function [5], relief of lumbar and pelvic pain [1], a shorter delivery time [6], less likelihood of cesarean section [7], and, to some extent, improved psychological well-being [8]. A randomized controlled trial that included 300 overweight or obese Chinese women with uncomplicated singleton pregnancies found that initiation of cycling exercise for at least 30 min three times per week during the first trimester of pregnancy significantly decreased the amount of gestational weight gain and the risk of GDM with no increase in the risk of preterm birth [4]. Prenatal exercise also has some benefits for the fetus; for example, facilitating neurological development [9] and reducing the risk of obesity at birth and in early childhood [10].

Considering the physiological and psychological benefits of engaging in regular prenatal exercise, the guidelines published by the World Health Organization (WHO) [2], the American College of Obstetricians and Gynecologists (ACOG) [1], the Society of Obstetricians and Gynecologists of Canada (SOGC), the Canadian Society for Exercise Physiology (CSEP) [11], the Department of Health and Social Care, UK [12], and the Australian Government Department of Health [13] all recommend that at least 150 min of moderate-intensity PA per week during pregnancy should be maintained when there are no pregnancy-related contraindications or complications. However, pregnant women are often physically inactive, with many who were previously active choosing to become inactive during pregnancy [14]. Compared with the PA level before conception, the level of PA during pregnancy is expected to be much lower and take a relatively single form. Recent studies showed that the rate of physical inactivity among pregnant women ranged from 27.2 to 88.9% [15–19]. In Brazil, Galliano et al. evaluated the PA level in 2,706 pregnant women with GDM in the second or third trimester using the International Physical Activity Questionnaire (IPAQ) and found that only 34.3% reached the recommended levels [18]. Studies in other countries have yielded similar results [17, 20]. Several studies have investigated PA in pregnant women in China and found that the proportion of women meeting the recommendations ranged from 11% in Tianjin [16] to 57.1% in Chengdu [21]. This wide variation in PA reflects differences in geographic region, local culture, and assessment methods. Lü et al. used 600 metabolic equivalent (MET) min/week as the cut-off value to define adequate PA in 2,485 pregnant women in China and found that only 32.7% of women in the first trimester and 55.3% in the second or third trimester engaged in an adequate amount of PA [22]. However, light-intensity PA was included when calculating MET in their study, which is different from the PA recommendations by the international guidelines. Therefore, the variable findings in these studies may reflect use of different measurement tools and non-standard evaluation methods.

Factors that affect PA in pregnant women are complex and include individual demographic, physiologic, psychologic, and lifestyle factors, as well as social support and environmental factors. Researchers have showed that pregnant women who are younger, less well educated, and unemployed and those with multiple children and a lower income are less likely to engage in an adequate amount of PA [16, 17, 23, 24]. Obstetric variables associated with PA in pregnancy include history of miscarriage, parity, and exercise and smoking status before pregnancy [25–27]. Even though there is no scientific evidence linking prenatal exercise with adverse outcomes, some obstetric caregivers and pregnant women continue to be skeptical about the safety of prenatal exercise.

Chinese families tend to comply with the traditional philosophy of protecting the fetus from miscarriage by advising pregnant women to rest as much as possible, particularly in the first trimester [28]. However, younger women often receive modern health care-based PA recommendations and have to strike a balance between conflicting concepts of rest and activity during pregnancy, which may result in more women actively engaged in walking rather than PA of moderate intensity [22]. There are still no specific PA guidelines for pregnant Chinese women, and little is known about the PA status of these women, especially in Shanghai, a modern metropolitan city in the east of China. Factors that affect the willingness of pregnant women to engage in an adequate amount of exercise is important to inform future interventions that can improve levels of PA during pregnancy. Therefore, the aims of this study were to investigate PA levels among pregnant women in Shanghai and to identify factors that influence the prevalence of physical inactivity.

## Methods

### Study design and participants

A cross-sectional study was conducted from May 2021 to March 2022 at a tertiary obstetrics and gynecology hospital in Shanghai, China. All pregnant women who had registered and received routine prenatal care in the obstetrics department at the hospital were approached and assessed for their eligibility by two researchers. Women were eligible to participate in the study if they were aged 18 years or older, able to read and understand questions, and able to provide written informed consent. Women with serious comorbidity (e.g., cardiovascular disease, anemia, or asthma), poor mental health status, or a physical disability were excluded.

### Ethical approval

The study protocol was approved by the ethics committee of the hospital (approval number 202123). All study participants provided written informed consent after receiving a verbal and written explanation about the purpose of the study and its procedures and being informed that they could withdraw from the study at any time for any reason.

### Sampling method and sample size

Convenient sampling approach was used to recruit pregnant women who had registered in the hospital. The sample size was estimated by the formula [29] with a 95% confidence level, a margin of error of 3%, and the prevalence of physical inactivity during pregnancy of 42.9% [21] (*p* = 0.429) as follows:

$$\mathrm{p}=0.429,\mathrm{ q}=1-\mathrm{p}=0.571,\mathrm{ e}=0.03, {\mathrm{z}}_{\mathrm{\alpha }}=1.96$$

$$\mathrm{So},\mathrm n=\frac{z_{\mathrm\alpha}^2\times pq}{\mathrm e^2}=\frac{\left(1.96\right)^2(0.429)(0.571)}{{(0.03)}^2}\approx1046$$

Addition of a further 10% attrition rate resulted in a required sample size of at least 1151. Finally, 1636 participants were enrolled in the study. We recruited more participants than the calculated sample size because more pregnant women than expected showed an interest in our research. Thus, when the time limit (time saturation) was reached, more participants were included [30].

### Measures

#### Main outcome

The International Physical Activity Questionnaire Short Form (IPAQ-SF) has been recommended as an effective method for assessment of the PA level in the general population, including pregnant women [31]. Different types of activity are assigned different MET values based on a standard reference [32]. One MET is equivalent to the energy expenditure of sitting 1 h quietly [33]. There are seven items in the IPAQ-SF [32], six of which are related to the frequency and duration of PA, including walking (3.3 MET), moderate-intensity PA (4.0 MET) such as yoga, Taichi, bicycling at a regular pace and playing table tennis, and vigorous-intensity PA (8.0 MET) such as swimming, running, rope skipping and aerobics. Total energy expenditure was figured out by summing up the MET scores from the energy expenditure of each activity in the past week.

#### Covariates and other measurements

Sociodemographic, behavioral, obstetric, and social support data were collected using a structured questionnaire that was developed in consultation with experts in the field and pilot tested in 10 respondents before the survey. The sociodemographic characteristics consisted of age, ethnicity, level of education, current employment status, personal monthly income, and pre-pregnancy body mass index (BMI), which was calculated as body weight divided by the square of height. Using the Chinese classification, women with a BMI < 18.5 kg/m^2^ are considered underweight, those with a BMI of 18.5–23.9 kg/m^2^ as normal weight, those with a BMI of 24–27.9 kg/m^2^ as overweight, and those with a BMI ≥ 28 kg/m^2^ as obese [34]. The behavioral characteristics before pregnancy are briefly described below. Regular exercise before pregnancy was defined as having consciously engaged in walking, cycling, yoga, running, or other PA for at least 30 min/week in the preconception period [35]. Pre-pregnancy smoking was defined as daily or intermittent smoking and passive smoking was defined as breathing second-hand tobacco smoke for more than 15 min a day in the 3 months before conception. Alcohol consumption before pregnancy was defined as drinking at least half bottle of beer, 40 mL of white wine, or 125 mL of red wine in a month. The obstetric parameters included history of spontaneous abortion, parity, stage of pregnancy, singleton pregnancy, nausea and vomiting during pregnancy, measures taken to prevent miscarriage, and GDM. The last 3 questions in the structured questionnaire were about social support. We collected information about social support by asking whether the participants had support from family members, friends and medical workers, which were sourced from the Social Support Rating Scale [36] and adapted for our survey.

Prenatal sleep quality was measured using the Chinese version of the Pittsburgh Sleep Quality Index (PSQI), which is the most frequently used generic measure of sleep in the research and clinical settings and has strong reliability and validity [37]. The PSQI includes 19 self-rated questions, which are divided into seven domains, including subjective sleep quality, sleep latency, sleep duration, sleep efficiency, sleep disturbances, use of sleeping medication, and daytime dysfunction. The score in each domain of the PSQI ranges from 0 to 3 and the total score ranges from 0 to 21. A total PSQI score > 5 indicates poor sleep quality and a total score ≤ 5 is regarded as good sleep quality [38].

Prenatal anxiety status was assessed using the Chinese version of the Self-rating Anxiety Scale, which contains 20 items and is scored by calculating the frequency of symptoms corresponding to anxiety in the past week. Each item is scored on a 4-point scale ranging from 1 to 4; the scores for the 20 items are then added to obtain a rough score, which is then multiplied by 1.25 and the integer component taken to derive the standard score. The cut-off score for this scale is 50 points; a score of 50–59 is classified as mild anxiety, 60–69 as moderate anxiety, and > 70 as severe anxiety [39].

Prenatal symptoms of depression were measured by the Edinburgh Postnatal Depression Scale, which is considered to have acceptable reliability, validity and applicability as a screening tool for depression during pregnancy [40]. This scale contains 10 items, each of which is scored from 0 to 3, with higher scores indicating a higher likelihood of perinatal depression. In general, a cut-off score of 10 or higher is recommended for detection of potential depression in Asian pregnant women [41].

### Statistical analysis

The characteristics of the participants were presented as descriptive statistics. Continuous variables were summarized as the mean ± standard deviation if normally distributed and as the median (interquartile range) if not. Categorical variables were shown as the frequency (percentage). The dependent variable in this study was physical inactivity, which was defined as total energy expenditure < 600 MET min/week. A binary logistic regression model was used to identify factors associated with physical inactivity in pregnant women. Adjusted odds ratios (ORs) and 95% confidence intervals (CIs) were calculated. The steps used to identify these factors were as follows. First, we used backward elimination to remove variables with a *p*-value > 0.2. Second, considering maternal age, level of education, pre-pregnancy BMI, parity, and history of spontaneous abortions to be clinically relevant or related to the level of PA in previous studies [20, 25, 26], we also added these five variables into the model. Finally, maternal age, level of education, current employment status, personal monthly income, pre-pregnancy BMI, regular exercise before pregnancy, alcohol consumption before pregnancy, history of spontaneous abortions, parity, stage of pregnancy, nausea and vomiting during pregnancy, and prenatal sleep quality were entered as independent variables into the logistic regression model. All statistical analyses were performed using the SPSS software package (version 25.0; IBM Corp., Armonk, NY, USA). A *p*-value of < 0.05 was considered statistically significant.

## Results

In total, 1636 women were enrolled in the study. Twenty-two of the women did not meet the inclusion criteria, 64 dropped out during the study period, and 35 returned invalid questionnaires (incomplete or with inconsistent responses), leaving data for 1515 participants for analysis.

### Characteristics of the participants

Table 1 presented the sociodemographic, behavioral, obstetric and social support characteristics of the study participants. The average age was 30.6 ± 3.7 years, and 18.1% of the women were overweight or obese according to the Chinese BMI classification. The socioeconomic status of the majority of the participants was good, with 75.7% having a personal monthly income of more than 10,000 yuan; 71.7% of the women were still working. More than half of the women (55.5%) undertook regular exercise before pregnancy. In total, 13.1% of the women reported tobacco exposure before pregnancy. Most of the women were nulliparous and 79.3% experienced severe nausea and vomiting during pregnancy. The questionnaires were completed by 668 women in the first trimester, 389 in the second trimester, and 458 in the third trimester. Poor prenatal sleep quality was a common complaint. Overall, the participants were well-educated with 92.8% having completed a college degree.

**Table 1:** Characteristics of the participants (*n* = 1515)

Variables	Frequency
Age (years), n (%)	
< 25	60 (4.0)
25–29	560 (37.0)
30–34	673 (44.4)
≥ 35	222 (14.7)
Ethnicity, n (%)	
Han	1491 (98.4)
Minorities	24 (1.6)
Level of education, n (%)	
High school or less	109 (7.2)
College	1002 (66.1)
Professional or graduate	404 (26.7)
Current employment status, n (%)	
Does not work	428 (28.3)
On duty	1087 (71.7)
Personal monthly income, n (%)^a^	
< ¥10,000	368 (24.3)
≥ ¥10,000	1147 (75.7)
Pre-pregnancy body mass index, n (%)	
Underweight	213 (14.1)
Normal weight	1029 (67.9)
Overweight	225 (14.9)
Obese	48 (3.2)
Family support, n (%), yes	1452 (95.8)
Friends support, n (%), yes	1425 (94.1)
Medical workers support, n (%), yes	1338 (88.3)
Regular exercise before pregnancy, n (%), yes	674 (44.5)
Active or passive smoking before pregnancy, n (%), yes	198 (13.1)
Alcohol drinking before pregnancy, n (%), yes	150 (9.9)
History of spontaneous abortions, n (%), yes	236 (15.6)
Parity, n (%)	
Primiparous	1233 (81.4)
Multiparous	282 (18.6)
Current stage of pregnancy, n (%)	
First trimester: ≤ 13 + 6 weeks	668 (44.1)
Second trimester:14–27 + 6 weeks	389 (25.7)
Third trimester: ≥ 28 weeks	458 (30.2)
Singleton pregnancy, n (%), yes	1499 (98.9)
Nausea and vomiting during pregnancy, n (%), yes	1202 (79.3)
Measures taken to prevent miscarriage, n (%), yes	612 (40.4)
Gestational diabetes, n (%), yes	58 (3.8)
Poor prenatal sleep quality, n (%), yes	639 (42.2)
Prenatal anxiety, n (%), yes	114 (7.5)
Prenatal depression, n (%), yes	391 (25.8)

^a^One Chinese Yuan (¥) = US $0.1492

### Physical activity levels of the participants

Table 2 showed the PA level overall during pregnancy and according to intensity. The median energy expenditure on total PA was 693 MET min/week. Overall, 129 women did not report any type of PA. Walking as the main form of PA accounted for 92.7% of total MET. The prevalence of physical inactivity was 47.5%, and only 2.8% of women achieved the goal of at least 150 min of moderate-intensity PA per week. Furthermore, 94.6% of the women who reported sufficient PA did not meet the guideline of more than 150 min of moderate-intensity PA per week (Table 3).

**Table 2:** Physical activity characteristics of the participants (*n* = 1515)

Physical activity	N (%)	Median (IQR)^b^	MET ratio (%)^c^
Energy consumption (MET-min/week)^a^			
Total activity	1515 (100.0)	693 (297, 990)	100.0
Light-intensity activity (walking)	1381 (91.2)	693 (396, 990)	92.7
Moderate-to vigorous-intensity activity	118 (7.8)	480 (240, 942)	7.3
Reported no physical activity	129 (8.5)	—	—
Physical inactivity			
No	796 (52.5)	—	—
Yes	719 (47.5)	—	—
Meeting the guideline			
No	1472 (97.2)	—	—
Yes	43 (2.8)	—	—

^a^*MET* Metabolic equivalent

^b^*IQR* Interquartile range

^c^MET ratio: The contribution of the metabolic equivalent of each intensity of activity to the total physical activity metabolic equivalent

**Table 3:** Prevalence of women meeting the international physical activity guidelines according to physical inactivity status (*n* = 1515)

Categories	PA^a^ < 600 MET min/week (*n* = 719)	PA^a^ ≥ 600 MET min/week (*n* = 796)
Do not meet the guideline, n (%)	719 (100.0)	753 (94.6)
Meet the guideline, n (%)	0 (0.0)	43 (5.4)

^a^*PA* Physical activity

### Factors related to physical inactivity during pregnancy

The results of the multivariable binary logistic regression analysis were presented in Table 4. Pregnant women with an individual income of more than 10,000 yuan per month (adjusted OR 0.648, 95% CI 0.505–0.831) were less likely to be physically inactive than those with a lower income. Moreover, regular exercise before conception contributed to maintaining adequate PA during pregnancy (adjusted OR 0.575, 95% CI 0.464–0.711). However, the physical inactivity rate was significantly higher in the first trimester than in the second trimester (adjusted OR 0.534, 95% CI 0.411–0.693) or third trimester (adjusted OR 0.615, 95% CI 0.470–0.806). Women who had nausea and vomiting during pregnancy were 1.307 times more likely to be physically inactive than those who did not have these symptoms (adjusted OR 1.307, 95% CI 1.002–1.705).

**Table 4:** Binary logistic regression analysis of the factors associated with physical inactivity

Variables	B	OR^a^	95% CI^b^	*P* value
Age (years)				
< 25	—	1.000	(reference)	—
25–29	0.299	1.348	0.771–2.358	0.295
30–34	0.359	1.432	0.819–2.502	0.208
≥ 35	0.323	1.381	0.750–2.544	0.300
level of education				
High school or less	—	1.000	(reference)	—
College	–0.148	0.862	0.570–1.301	0.481
Professional or graduate	0.024	1.024	0.655–1.594	0.916
Personal monthly income^d^				
< ¥10,000	—	1.000	(reference)	—
≥ ¥10,000	–0.434	0.648	0.505–0.831	0.001 ^c^
Regular exercise before pregnancy				
Yes	–0.554	0.575	0.464–0.711	< 0.001 ^c^
Alcohol drinking before pregnancy				
Yes	0.232	1.261	0.885–1.798	0.199
Pre-pregnancy body mass index				
Underweight	—	1.000	(reference)	—
Normal weight	0.028	1.029	0.756–1.399	0.857
Overweight	–0.294	0.745	0.503–1.103	0.142
Obese	0.146	1.157	0.603–2.219	0.661
History of spontaneous abortions				
Yes	–0.013	0.930	0.738–1.319	0.930
Current stage of pregnancy				
First trimester: ≤ 13 + 6 weeks	—	1.000	(reference)	—
Second trimester:14–27 + 6 weeks	–0.627	0.534	0.411–0.693	< 0.001 ^c^
Third trimester: ≥ 28 weeks	–0.485	0.615	0.470–0.806	< 0.001 ^c^
Nausea and vomiting during pregnancy				
Yes	0.268	1.307	1.002–1.705	0.049 ^c^
Poor prenatal sleep quality				
Yes	–0.199	1.220	0.984–1.513	0.069

^a^*OR* Odds ratio

^b^*95% CI* 95% confidence interval

^c^*P* < 0.05

^d^One Chinese Yuan (¥) = US $0.1492

## Discussion

This study evaluated physical inactivity and related factors in pregnant women in Shanghai, China. We found that 47.5% of our study participants do not engage in sufficient PA during pregnancy, with an average of only 287.78 MET min/week, which is reasonably consistent with the figure of 44.75% reported for the 15 provinces in China [22]. Use of the same criteria with a total energy expenditure of < 600 MET min/week to define physical inactivity may account for these findings. However, the proportion of pregnant women in a study from Serbia [15] who do not have adequate PA was found to be slightly lower despite using the same classification standard, and may reflect a difference in the trimesters studied or a difference in the study populations. In a study that used the Prenatal Physical Activity Questionnaire, 57.1% of pregnant women reached the recommended level (≥ 150 min of moderate-intensity activity per week) in Chengdu [21], whereas only 11% fulfilled the recommendations during pregnancy (≥ 2 h of moderate-intensity exercise per week) in Tianjin [16]. Studies from other countries, no matter the developed countries, including the United States, Norway and Ireland [19, 42, 43], the developing countries including Brazil and Vietnam [24, 44], and underdeveloped countries in South Africa [20] consistently reported lower PA levels in pregnant women, despite using different measurement tools and evaluation methods.

In our study, only 2.8% of the pregnant women achieved the level of prenatal PA recommended by the international guidelines. In fact, moderate-intensity activity was assigned a MET value of 4 in this investigation, to some extent, the international guidelines of at least 150 min of moderate-intensity activity per week is equivalent to 600 MET min/week. However, more than ninety percent of our study participants who reported sufficient PA of ≥ 600 MET min/week did not meet the international guidelines. The main reason for such a huge difference between the two results may be that walking, a light-intensity activity, was the major form of PA and accounted for 92.7% of total MET values in our population. It has been suggested that some health care workers have conservative views about PA during pregnancy and question the safety of moderate-to-vigorous intensity PA, which may result in recommendations for lighter PA during pregnancy [45]. Our finding that PA during pregnancy consisted of walking which accounted for a relatively large proportion of PA in our study is similar to that of studies in Ethiopia and Poland [17, 27]. In our study, walking was assigned a MET value of 3.3 and included in calculating the result of PA level to define sufficient PA. However, walking is excluded from the international guidelines, which focus only on moderate or vigorous intensity PA. And without any doubt, moderate or vigorous intensity activities can provide substantial health benefits; for example, pregnant women engaged in at least 600 MET min/week of moderate PA (e.g., 140 min of brisk walking and stationary cycling) can reduce their risk of GDM or gestational hypertension by at least 25% [46]. Light-intensity activities, such as walking, can have health benefits too, but the greatest benefit often occurs when sedentary behavior is replaced with moderate or vigorous intensity PA [47]. Therefore, we should suggest a gradual increase in the amount and intensity of activity; for example, increasing their walking pace and choosing exercise that is more acceptable, such as brisk walking, to promote the moderate or vigorous intensity PA recommended by the guidelines.

We used a multivariable logistic regression model to identify factors associated with physical inactivity during pregnancy in Shanghai and found that women who had engaged in regular exercise before pregnancy were more likely to have a higher PA level during pregnancy than women who had not, which is consistent with studies from Arba Minch Town in Ethiopia [25], Campinas in Brazil [24], data from the US Centers for Disease Control and Prevention [19] and a study performed in Chengdu in China [21]. This finding indicates that starting exercise before conception may help women to achieve adequate PA during pregnancy and underscores the importance of lifestyle changes before pregnancy. Of note, a prospective study from Spain found that nearly half of 1,175 pregnant women engaged in sufficient PA before pregnancy but did not maintain their PA level during pregnancy [14]. This may be attributed to physical discomfort during pregnancy, especially nausea and vomiting [48], which is consistent with our findings, in addition to low back pain, fatigue, fear of injury, and uncertainty about the safety of exercising during pregnancy [49].

In our study, women with higher incomes were less likely to have a low PA level during pregnancy, which is in line with the reports by Galliano et al. and Yu et al. [18, 23]. The study in Serbian women found that inadequate PA during pregnancy was more marked in low-income families, which could reflect a lack of leisure time for exercise [15]. However, unlike other studies [26, 50], we did not find any relationship between physical inactivity status during pregnancy and other social factors, such as social support or educational level. We also found a decrease in the likelihood of physical inactivity as pregnancy advanced from the first trimester to the second and third trimesters. A cross-sectional study of 1077 Chinese pregnant women found that pregnant women in the third trimester were more likely to meet the international guidelines than those in the early stages of pregnancy [21]. Likely explanations for this finding include the traditional Chinese practice of protecting the fetus from miscarriage by resting and physical discomfort, particularly morning sickness. However, as mentioned above, the available evidence indicates that interventions to improve the PA level during late pregnancy should be implemented during early pregnancy or even before pregnancy. The cohort study showed that women with adequate PA levels in early pregnancy were more likely to engage in adequate PA in mid-to-late pregnancy [22]. Similarly, Okafor and Goon showed that women who started PA in the first trimester were more active and more likely to meet the guideline than those who started PA in the second trimester [20].

Although various factors including socioeconomic status, PA levels before pregnancy, as well as physiological changes during pregnancy, may affect the ability to engage in PA, there is limited qualitative information in the literature on how these factors affect women’s PA during pregnancy. More qualitative or mix-method studies are needed in the future to improve our understanding about women’s willingness or otherwise to engage in PA during pregnancy and inform interventions in clinical practice. What’s more, walking was the predominant form of PA in our population. It is worth thinking about the effects of different intensities of PA, such as walking or moderate-intensity activities, on pregnancy outcomes or long-term health effects of mothers and offspring. Therefore, further comparative studies are needed.

The results of our study provided some basic information of PA status among Chinese pregnant women, which can also help health professionals as well as policymakers to take tailored measures to promote adequate PA among this population. To be specific, first, early intervention is needed, especially for women who do not exercise regularly before pregnancy. Second, as to the low-income pregnant women, we advocate the government and relevant departments to implement corresponding policies, such as providing free consultations, sports grounds and brochures, to alleviate the impacts of poor economic conditions. In addition, it is necessary to provide systematic training of PA knowledge for medical workers, which is crucial for them to provide evidence-based scientific recommendations of PA for pregnant women.

### Limitations

This study had several limitations. First, the PA data were self-reported using the IPAQ, which introduces the possibility of reporting bias. More accurate data on the amount and frequency of PA during pregnancy would be obtained by use of electronic activity monitors, such as wearable bracelets, accelerometers, or pedometers. Second, the study had a cross-sectional design, which meant that we could not obtain information on changes in the PA level as the women moved through the trimesters. Therefore, prospective studies are needed in the future. Third, our study participants were from only one hospital in Shanghai and may not be representative of the entire city. However, despite these limitations, this study provides some useful information concerning physical inactivity during pregnancy in Shanghai that can be used for comparative purposes in future studies.

## Conclusion

The prevalence of physical inactivity among pregnant women in China remains high, particularly among women with a lower monthly income, those who are not engaged in regular exercise before pregnancy, and those who experience nausea and vomiting during pregnancy. More efforts should be taken to help these women to overcome the barriers to prenatal PA. Furthermore, we need a better understanding of the dynamic changes in PA that occur during pregnancy and whether these may be affected by the coronavirus pandemic, which is likely to continue into the future. Further researches including a combination of quantitative analysis and qualitative method are needed to obtain more information on how to promote PA during pregnancy.

## Data Availability

The data that support the findings of this study can be obtained by contacting the corresponding author Na Wang, upon reasonable request.

## Abbreviations

PA: Physical activity
GDM: Gestational diabetes mellitus
WHO: World Health Organization
ACOG: American College of Obstetricians and Gynecologists
SOGC: Society of Obstetricians and Gynecologists of Canada
CSEP: Canadian Society for Exercise Physiology
IPAQ: International Physical Activity Questionnaire
MET: Metabolic equivalent
IPAQ-SF: International Physical Activity Questionnaire Short Form
TEE: Total energy expenditure
BMI: Body mass index
PSQI: Pittsburgh Sleep Quality Index
ORs: Odds ratios
CIs: Confidence intervals
IQR: Interquartile range
